# High Accuracy Heartbeat Detection from CW-Doppler Radar Using Singular Value Decomposition and Matched Filter

**DOI:** 10.3390/s21113588

**Published:** 2021-05-21

**Authors:** Yuki Iwata, Han Trong Thanh, Guanghao Sun, Koichiro Ishibashi

**Affiliations:** 1 Graduate School of Informatics and Engineering, The University of Electro-Communications (UEC), Tokyo 182-8585, Japan; guanghao.sun@uec.ac.jp (G.S.); ishibashi@uec.ac.jp (K.I.); 2 School of Electronics and Telecommunications, Hanoi University of Science and Technology (HUST), Hanoi 100000, Vietnam; thanh.hantrong@hust.edu.vn

**Keywords:** continuous wave-Doppler radar, non-contact vital signs measurement, matched filtering, singular value decomposition

## Abstract

Heart rate measurement using a continuous wave Doppler radar sensor (CW-DRS) has been applied to cases where non-contact detection is required, such as the monitoring of vital signs in home healthcare. However, as a CW-DRS measures the speed of movement of the chest surface, which comprises cardiac and respiratory signals by body motion, extracting cardiac information from the superimposed signal is difficult. Therefore, it is challenging to extract cardiac information from superimposed signals. Herein, we propose a novel method based on a matched filter to solve this problem. The method comprises two processes: adaptive generation of a template via singular value decomposition of a trajectory matrix formed from the measurement signals, and reconstruction by convolution of the generated template and measurement signals. The method is validated using a dataset obtained in two different experiments, i.e., experiments involving supine and seated subject postures. Absolute errors in heart rate and standard deviation of heartbeat interval with references were calculated as 1.93±1.76bpm and 57.0±28.1s for the lying posture, and 9.72±7.86bpm and 81.3±24.3s for the sitting posture.

## 1. Introduction

Cardiopulmonary activity-related information, such as heart rate (HR) and respiratory rate, is known to be an effective indicator for monitoring the health and mental state of a subject. The CW-Doppler radar sensor (CW-DRS) is a non-contact cardiopulmonary information collection technique that has garnered significant attention in diverse research areas [1,2,3,4]. Whereas electrocardiograms (ECGs) and photoplethysmography (PPG) require a sensor to be attached to the subject [5,6], a CW-DRS allows non-contact signal acquisition over clothing [7,8,9]. Therefore, CW-DRSs have been investigated for application in situations where contact with the subject is hazardous for the experimenter or when restraining the subject is difficult; examples include screening systems for infectious diseases at airports to prevent the spread of infectious diseases [10,11,12,13], health monitoring of patients with burns or skin lacerations during disasters [14,15], and biometric monitoring while driving [16]. Furthermore, a technique to capture more minute heart rate information, such as the P-wave and Q-wave, using CW-DRS has been proposed in recent years [17,18]. The use of CW-DRSs in biometric applications is expected to increase in the future.

As CW-DRSs acquire small vibrations produced on the surface of the chest by cardiac activity, they are susceptible to chest vibrations that are unrelated to heartbeats, such as breathing and body movements, as well as interference from other people [19,20,21]. When attempting to separate heart rate and respiratory waveforms, the fact that the frequency characteristics of each signal are different is often considered. However, the intensities of the second and third harmonics of the respiratory waveform in the spectrum of the heartbeat are comparable to the intensity of the heartbeat signal [22,23]. Therefore, this problem cannot be solved by simple signal processing, such as using a bandpass filter or Fourier transform. Yang et al. [13] proposed a HR measurement technique using a bandpass filter and CW-DRS to develop a dengue fever quarantine system at airports. The author conducted a large-scale evaluation experiment with 410 subjects; however, 90% of the data could not be used for analysis due to the effects of respiratory harmonics and random body movements (RBMs) mentioned above.

Hence, researchers have proposed various approaches using more complex signal processing methods [24,25,26,27,28]. Petrovic et al. [24] focused on the harmonic vibration of the chest surface and proposed extracting the harmonic signal of heartbeats from the vibration of a chest surface acquired using a CW-DRS with a bandpass filter. In this method, it is assumed that respiration does not exist in the harmonic region of the heartbeat. In addition, Saluja et al. [25] used a gamma filter as a machine-learning approach to remove the harmonics of breathing. These methods were developed to eliminate the harmonics of respiration and do not consider body motion. However, in contrast to respiration, body motion does not occupy a specific frequency band, and its amplitude is extremely large. Therefore, pre-informative approaches, such as frequency filters and gamma filters, are not suitable for body motion removal. In contrast, an adaptive approach using a matched filter (MF) has been proposed to address body motion. An MF emphasizes the target signal by convolving it with the input signal as a template when the shape of the waveform to be acquired is known. This technique is similar to correlation detection in receivers and template matching in machine learning. To extract heartbeats from chest vibration using MF, a template of the heartbeat must be prepared. Many researchers proposed algorithms to adaptively generate a template from the output of radar [26,27,28]. In other studies [26,27], the template was created using polynomial approximation to remove breathing and body motion from measurement signals extracted in a 2 s time window. Lv et al. [26] evaluated the effectiveness of the proposed method through simulations and actual measurements and successfully eliminated the effects of harmonics of breathing and body motion. However, they remarked that this method requires a 2 s visual selection, where no body motion is present, to enable a template to be created. Izumi et al. [27] proposed a technique that combines an MF with the Burg method, in which the time–frequency characteristics of measurement signals are measured. This method requires a high sampling frequency and sufficient data length to generate a template in the time–frequency domain. Therefore, it is desirable to develop a signal processing method to extract highly accurate and robust heart rate signals from time-domain signals obtained with short data lengths and low sampling frequencies.

Herein, we propose SVD+MF method that combines an MF and singular value decomposition (SVD) as a signal processing algorithm to detect heart rate information with high accuracy from chest surface vibrations of short data length (less than 30 s). In this method, a heartbeat signal recovery technique involving an MF is used, and singular vectors are applied in the trajectory matrix of the measured signal as templates. The trajectory matrix is a matrix composed of a partial time series of time-domain signals, and it is primarily used for outlier detection and signal decomposition in signal processing [29]. The effectiveness of the proposed method in detecting heartbeats depends on the data length of the partial time series and the singular vector used as a template. In this study, we investigated the relationship between these parameters and the accuracy of heartbeat detection using the vibration of the chest surface modeled on a computer. In addition, we evaluated the effectiveness of our method in various situations by conducting measurements in two different postures: lying and sitting.

## 2. Basic Formula of CW-Doppler Radar

**Figure 1:**
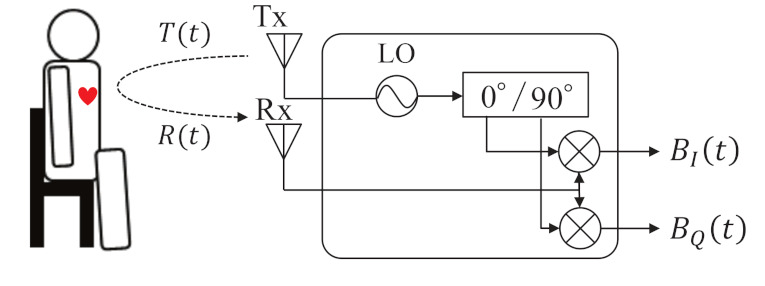
Fundamental mechanism of CW-Doppler radar.

Figure 1 shows the basic structure of the CW-DRS for vital sign detection [1,7,8,9]. The transmitter Tx transmits a continuous wave $T\left(t\right)=A_{T}\cos\left[2\pi ft+\phi \left(t\right)\right]$ toward the human body surface, where $A_{T}$ is the amplitude of the transmitted signal, *f* is the carrier frequency, and ϕ(t) is the phase noise. Based on the Doppler principle, the frequency of reflected wave changes according to the body surface motion, which is composed of respiration $x_{r}$, heartbeat $x_{h}$, and RBM $x_{m}$. Therefore, the receiver Rx receives the reflected wave R(t), which is expressed as follows:

(1) $$\begin{array}{c} R\left(t\right)=A_{R}\cos\left[2\pi ft-\frac{4\pi d_{0}}{\lambda }-\frac{4\pi x(t)}{\lambda }+\phi \left(t-2d_{0}c\right)\right], \end{array}$$

where $A_{R}$ is the amplitude of the received signal, λ is the wavelength, *c* is the speed of light, $d_{0}$ is the initial distance between the CW-DRS and the body surface, and $x\left(t\right)=x_{r}+x_{h}+x_{m}$ is the displacement of human body surface. As shown in Figure 1, when the received signal R(t) is down-converted, two baseband signals are obtained. One is the in-phase signal $B_{I}\left(t\right)$, and the other is the quadrature phase signal $B_{Q}\left(t\right)$.

(2) $$\begin{array}{c} B_{I}\left(t\right)=A_{I}\cos\left[\frac{4\pi \{x\left(t\right)+d_{0}\}}{\lambda }+\theta +\Delta \phi \left(t\right)\right]\;, \end{array}$$

(3) $$\begin{array}{c} B_{Q}\left(t\right)=A_{Q}\sin\left[\frac{4\pi \{x\left(t\right)+d_{0}\}}{\lambda }+\theta +\Delta \phi \left(t\right)\right]\;, \end{array}$$

where $A_{I}$ is the amplitude of the in-phase signal, and $A_{Q}$ is the amplitude of quadrature phase signal, θ the phase constant, and Δϕ(t) phase noise. As these signals are associated with a “null point” problem, the detection of the variable object generally requires demodulation [30,31]. The arctangent, which is an effective method involving two orthogonal signals to demodulate the phase information, was adopted in this study. It is expressed as follows:

(4) $$\begin{array}{c} x\left(t\right)\approx \frac{\lambda }{4\pi }\arctan\left(\frac{B_{Q}\left(t\right)}{B_{I}\left(t\right)}\right)\;. \end{array}$$

Here, we assume that $A_{I}$ and $A_{Q}$ are equal, and θ and Δϕ(t) are small.

## 3. Proposed Method

**Figure 2:**
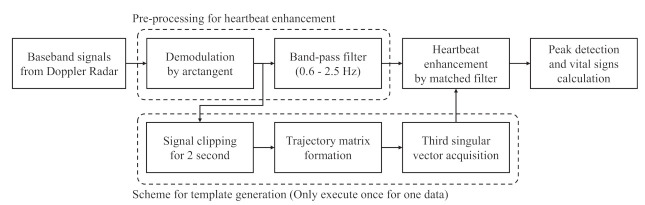
The diagram showing the proposed method for extracting cardiac information.

Figure 2 presents a diagram of the SVD+MF method for extracting cardiac information from baseband signals with high accuracy. First, the baseband signals, $B_{I}\left(t\right)$ and $B_{Q}\left(t\right)$, were demodulated by the arctangent method to extract phase information related to the displacement of the chest surface. Subsequently, the demodulated signal was forwarded to the bandpass filter (BPF) stage, which is the pre-processing for the heartbeat enhancement by matched filtering and the heartbeat template generation stage in the matched filtering. During pre-processing, the undesired frequency components are reduced from the demodulated signal by a BPF. In this study, our focus is on the cardiac frequency; therefore, we set the bandwidth from 0.6 to 2.5 Hz. Here, an IIR-type digital filter (order: 20) is used. Meanwhile, the template generation stage comprises three components: extraction of the 2 s data from the demodulated signal, formation of the trajectory matrix from the extracted signal, and calculation of the third singular vector by the SVD of the trajectory matrix. Subsequently, matched filtering was performed using the signal filtered by the BPF and the third singular vector to obtain an enhanced heartbeat waveform. Finally, the peaks in the enhanced heartbeat waveform were detected to calculate the heart rate information, such as the HR and standard deviation of heartbeat intervals (SDHI) from their intervals.

### 3.1. Heartbeat Enhancement by Matched Filtering

Matched filtering is a technique used to maximize the SNR of a signal in the presence of additive noise [32,33,34]. By convoluting the template signal with the input signal, the template signal hidden in the input signal can be identified, resulting in a significant improvement in the SNR. In our signal processing, the respiratory waveform included in the vibration of the chest surface is considered as an added noise to the heart rate signal. Therefore, if we can prepare a template for the heartbeat, then we can detect the heartbeat signal hidden in the vibration of the chest surface by the MF. The demodulated signal representing the chest surface vibration, expressed in Equation (4), can be rewritten in terms of the heartbeat $x_{heart}\left[n\right]$ and additive noise $x_{noise}\left[n\right]$ as follows:

(5) $$\begin{array}{c} x\left[n\right]=x_{heart}\left[n\right]+x_{noise}\left[n\right]. \end{array}$$

Here, $x_{noise}\left[n\right]$ contains white noise and body movement noise. Subsequently, the MF can recover $x_{heart}\left[n\right]$ by convolution of x[n] with the conjugate and time-reversed version of the template h[n], as follows:

(6) $$\begin{array}{c} x_{heart}\left[n\right]=x\left[n\right]*h^{*}\left[-n\right]. \end{array}$$

### 3.2. Template Generation by Singular Value Decomposition

When using an MF, a template with the maximum SNR should be used. According to the MF principle, a template should have at least one heartbeat cycle for heartbeat extraction [1]. In addition, considering that the heart rate waveform differs by the individual, we designed a scheme to adaptively form a template of the heartbeat waveform using part of the demodulated signal. The scheme comprises three processes, as shown in Figure 2. First, we define the 2 s of data clipped from the demodulated signal as a lagged vector, expressed as follows:

(7) $$\begin{array}{c} y_{i}={\left\{x_{i},x_{i+1},\ldots ,x_{i+L-1}\right\}}^{T}\;, \end{array}$$

where *L* is the data length of the lagged vector $y_{i}$. In this study, we set L=200 as the sampling frequency was set to 100 Hz. Subsequently, we defined a trajectory matrix X composed of the lagged vectors as

(8) $$\begin{array}{ccc} X & = & \left\{y_{1},y_{2},\ldots ,y_{M}\right\}\\ & = & \left(\begin{array}{cccc} x_{1} & x_{2} & \cdots & x_{L}\\ x_{2} & x_{3} & \cdots & x_{L+1}\\ \vdots & \vdots & \ddots & \vdots \\ x_{M} & x_{M+1} & \cdots & x_{M+L-1} \end{array}\right). \end{array}$$

Finally, we performed SVD on the trajectory matrix X, as follows:

(9) $$\begin{array}{c} X=\sum_{i=1}^{r}\sigma_{i}u_{i}v_{i}. \end{array}$$

Here, *r* represents the rank of the matrix X; $u_{i}$ and $v_{i}$ are the left and right singular vectors corresponding to singular values $\sigma_{i}$, respectively. In this method, the vector that most closely relates $v_{3}$ to the heartbeat among these right singular vectors is used as the template ***h*** for the MF.

## 4. Simulation Analysis

As the singular vector obtained by the SVD of the trajectory matrix was used as the template in the SVD+MF method, the characteristics of the template were determined by the length of the lagged vectors in the trajectory matrix and the corresponding singular values. Therefore, the effect of the proposed method was assumed to be affected by these parameters. However, it was difficult to adjust the parameters using the measured data as the heart rate waveform varied based on the individual being measured and the user’s mental state. Furthermore, the CW-DRS signal contained not only heart rate and respiration data, but also noise and distortions from the receiver. In this section, we evaluate the relationship between the proposed heartbeat enhancement method and the parameters by simulating the vibration of the chest surface.

### 4.1. Configuration of Heart Rate and Respiratory Signal Models

The surface of the human chest is known to vibrate slightly owing to cardiopulmonary activities such as heartbeat and respiration. In this study, the vibration m(t) of the human chest surface is defined as shown in Equation (10).

(10) $$\begin{array}{c} m\left(t\right)=m_{heart}\left(t\right)+m_{resp}\left(t\right)+w\left(t\right)\;. \end{array}$$

**Figure 3:**
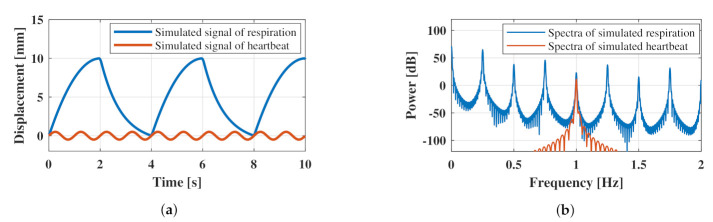
Simulated chest surface oscillations caused by breathing and heartbeat: (**a**) shows the signal in the time domain and (**b**) shows the spectrum in the frequency domain.

Here, $m_{heart}\left(t\right)$ and $m_{resp}\left(t\right)$ represent the modeled vibration of the chest surface caused by heartbeat and respiration, respectively; w(t) represents the white noise. Next, we describe the modeling of the heartbeat and respiration. Regarding the heartbeats, various studies have been conducted previously, where chest variability derived from the heartbeat was modeled [35,36,37]. The heartbeat waveform was represented by a sinusoidal pulse [35], half-wave sinusoidal pulse [36], Gaussian pulse [37], and a combination of two different pulses per beat [38]. One of the most significant differences between these models is whether the harmonics of the heartbeat waveform are considered. Harmonics were considered in the models presented in [37,38]; in particular, the model presented in [38] considered oscillations caused by the QRS complex and the T waves of the ECG waveform. As the algorithm proposed herein focuses on the fundamental wave of the heartbeat, the model in [35] based on the sine wave was used. Model respiration is covered in [38]. It has been suggested in [38] that the respiration model can be represented as follows:

(11) $$\begin{array}{c} {\hat{m}}_{resp}\left(t\right)=\left\{\begin{array}{cc} \frac{-K_{b}}{T_{i}T_{e}}t^{2}+\frac{K_{b}T}{T_{i}T_{e}}t, & t\in [0,T_{i}],\\ \frac{K_{b}}{1-e^{-\frac{T_{e}}{\tau }}}t^{2}\left(e^{-\frac{\left(t-T_{e}\right)}{\tau }}-e^{-\frac{T_{e}}{\tau }}\right), & t\in [T_{i},T] \end{array}\right. \end{array}$$

where $K_{b}$ is a model constant related to the amplitude adjustment of the respiratory waveform; $T_{i}$, $T_{e}$, and *T* represent the inspiration time, exhalation time, and respiratory cycle, respectively ($T=T_{i}+T_{e}$); τ is the time constant. Figure 3 shows examples of the simulated heartbeat and respiratory waveform generated using MATLAB.

The respiratory rate was set to 15 bpm (0.25 Hz) and the displacement was set to 10 mm. As shown in Figure 3a, the modeled respiratory waveform differed from a sinusoidal wave in terms of its shape between exhalation and inhalation. As shown in Equation (11), the exhalation part is represented by a quadratic polynomial, whereas the inhalation part is represented by an exponential function. Therefore, harmonics were included in the respiration model. Figure 3b shows the frequency characteristics of the respiration model obtained using the Fourier transform shown in Figure 3a. As presented in Figure 3b, the most dominant spectrum occurred at 0.25 Hz, which was the fundamental frequency of this model. In addition, focusing on the harmonics, we confirmed that the odd-order harmonic components exhibited higher powers than the even-order harmonic components. This result is consistent with that of a previous study [38]. To achieve this waveform, various parameters in Equation (11) are presented in Table 1.

### 4.2. Method for Determining Parameters

The parameters of the proposed method were determined using the vibration model of the chest surface, as defined in Equation (10). The parameters were the length of the lagged vector constituting the trajectory matrix in the proposed method and in which a singular vector was used. To determine these two parameters, we applied the proposal method to the chest vibration model while varying the parameters to obtain the parameter with the highest heart rate estimation accuracy. As the ratio of respiration to heart rate in the chest surface vibration can vary easily by individual and the environment, we selected signals with various signal-to-noise ratios (SNRs). In this study, the SNR is defined as

(12) $$\begin{array}{c} SNR=20\log_{10}\left\{\frac{P_{heart}}{P_{resp}+P_{noise}}\right\}\;\left[\mathrm{dB}\right], \end{array}$$

where $P_{heart}$, $P_{resp}$, and $P_{noise}$ indicate the intensities of $x_{heart}\left(t\right)$, $x_{resp}\left(t\right)$, and w(t), respectively. When sweeping the SNR in the simulation, the power of the heartbeat and white noise were constant, whereas the power of the respiratory waveform was varied. The root mean square error (RMSE) was used as an indicator to evaluate the effectiveness of the proposed method for heart rate estimation. In this study, the RMSE is expressed using the beat-to-beat interval (BBI) of the heartbeat as follows as

(13) $$\begin{array}{c} RMSE=\sqrt{\sum_{i=1}^{N}{\left(BBI_{estimate}\left[i\right]-BBI_{true}\left[i\right]\right)}^{2}}\;\left[s\right]\;, \end{array}$$

where $BBI_{estimated}$ and $BBI_{true}$ are the adjacent estimated and true BBIs of heartbeat, respectively.

### 4.3. Simulation Results

**Figure 4:**
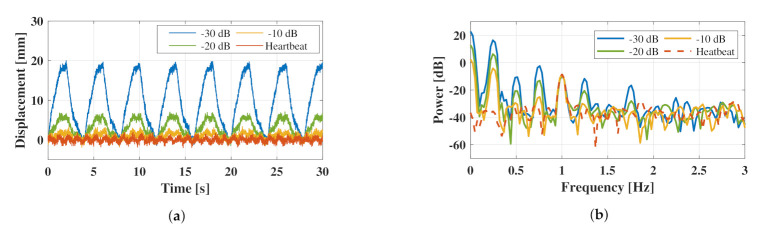
Vibration of the chest surface obtained by simulation: (**a**) shows the signal in the time domain and (**b**) indicates the spectrum by Fourier transform.

To determine the parameters of the proposed method, the simulated chest surface vibration was analyzed by sweeping the SNR in intervals of 10 dB from −30 to −10 dB. The model waveforms of the chest vibration at each SNR and the frequency characteristics obtained by the Fourier transform are shown in Figure 4. Figure 4a shows the time-domain signal of the chest vibration, whereas Figure 4b shows its frequency response.

The specific parameters for the simulation of the heartbeat and respiratory waveforms are listed in Table 2.

Figure 4b shows that the spectrum of the fundamental wave of the heartbeat occurred at approximately 1 Hz, and that the spectrum of respiration appeared over a wide range from 0.25 to 2 Hz. In the spectrum with only the heartbeat (0 dB), the heart rate was easy to estimate; however, in the other spectra, the heartbeat was suppressed by respiration. The −10 dB spectrum indicates that the peak of the spectrum at the fundamental frequency of respiration was comparable to the peak of the heartbeat. In such cases, separation techniques such as using frequency filters can be applied to solve the problem; however, the peak of the heartbeat and the respiratory harmonics exist in the same frequency band in the spectrum of −20 or −30 dB. Therefore, a more complex separation technique is required to extract weak heart rate signals from the respiratory waveforms.

**Figure 5:**
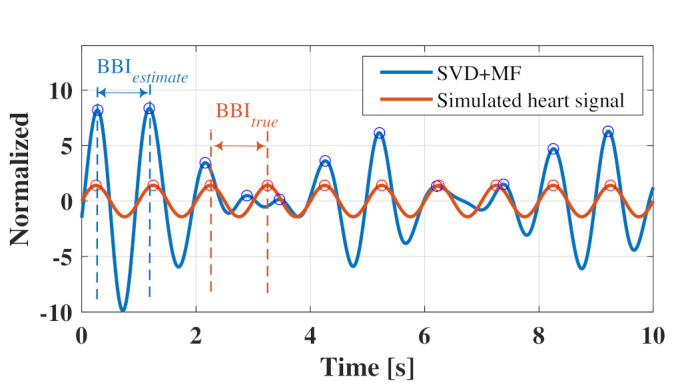
Example of a vibration model of a chest surface to which the proposed method was applied.

Figure 5 shows an example of the vibration model of the chest surface wherein the proposed method was applied to calculate the RMSE.

As shown in Figure 5, the peak of the waveform obtained by the proposed method generally coincided well with the peak of the heart rate signal. The RMSE was calculated using heart rate intervals $BBI_{estimate}$ and $BBI_{true}$, which were estimated from the peaks of the two waveforms.

**Figure 6:**
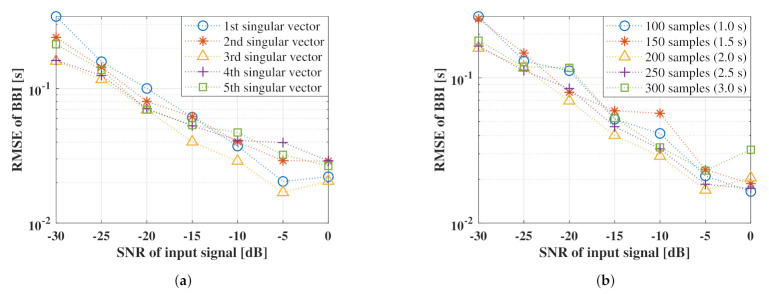
Accuracy evaluation of the proposed method for each parameter: (**a**) illustrates the comparison used the singular vectors, (**b**) shows comparison for the length of the lagged vector.

Figure 6 shows the relationship between the heart rate estimation accuracy calculated from the heart rate waveform obtained using the proposed method for each parameter and the SNR of the input signal. Figure 6a shows a comparison of the singular vectors used in the template of the proposed method, whereas Figure 6b shows a comparison of the length of the Lagged vector.

Figure 6a shows that a lower the SNR of the input signal resulted in a higher RMSE of BBI and a higher tendency for the accuracy to deteriorate. In other words, when the proposed method is applied to a low SNR signal, the respiratory waveform that remains unreduced will cause an error in the heart rate estimation. This effect is evident at approximately 3 s in Figure 5, where a peak mismatch occurred between the reference and estimated waveforms. Moreover, Figure 6a shows that the third singular vector indicated the highest accuracy over a wide range from 0 to −30 dB when a heart rate estimation was performed using each singular vector as a template. Subsequently, as shown in Figure 6b, the highest accuracy was obtained when 200 samples (2.0) were used as the data length of the Lagged vector. Therefore, it is considered optimal to use a lagged vector with a data length of 200 samples to form the trajectory matrix.

## 5. Experiment

### 5.1. Experimental Condition

In this study, we conducted two types of experiments with different subject postures. Consent was obtained from all the subjects. The first measurement condition involved a supine posture to evaluate the effect on data with small body movements. We irradiated a subject lying on a bed with a 24 GHz Doppler radar from the back and collected heart rate reference values via ECG simultaneously. Subsequently, we visually extracted six data points for 20 s without corruption by body movements. The second measurement was performed in the sitting posture, which was easily affected by body movements. In this experiment, the subject was irradiated from the front with a 24 GHz Doppler radar and reference values of the heart rate were obtained using PPG simultaneously. The subjects were 212 students from Vietnam National University, University of Engineering and Technology, and Hanoi University of Science and Technology.

**Figure 7:**
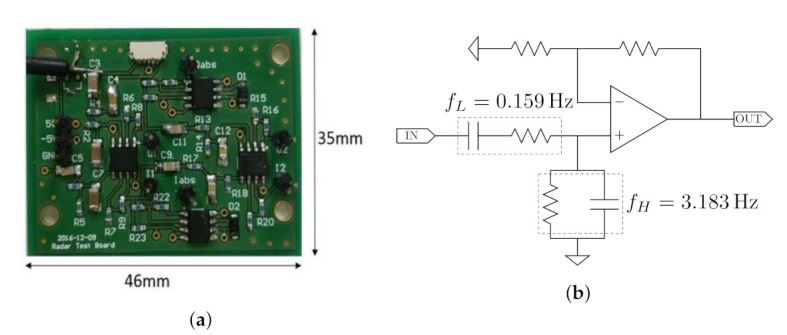
An (**a**) overview and (**b**) schematic of the developed circuit.

To measure the vibration on the surface of the chest, a commercially available 24 GHz Doppler radar-based moving object detection sensor, NJR4262J (New Japan Radio Co. Ltd., Tokyo, Japan), was used in this experiment, and its output was connected to an analog circuit developed for amplifying and filtering the signal. Figure 7 shows an overview of the developed circuit. The passband of the circuit was set between 0.159 and 3.183 Hz to eliminate undesired frequencies such as high-frequency noise. The output of the circuit was obtained using a commercial ADC converter USB-6008 (National Instruments Co., Austin, TX, USA). Its sampling frequency and voltage resolution were 100 Hz and 12 bits, respectively.

### 5.2. Description of Effectiveness of Proposed Method Using Actual Data

**Figure 8:**
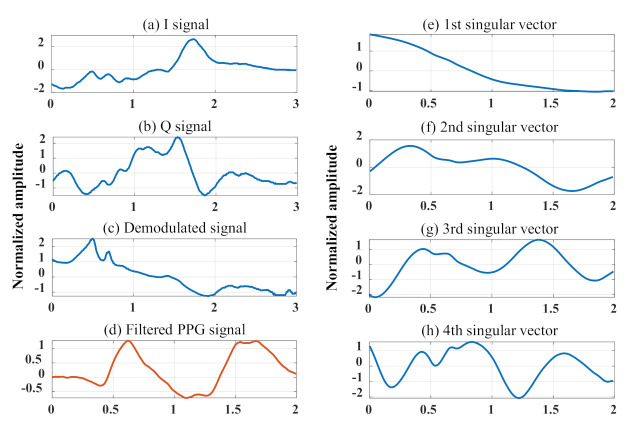
Examples of singular vector generation: (**a**,**b**) represent baseband signals from 24 GHz CW-DRS, (**c**) is the demodulated waveform by Arctangent, (**d**) shows the filtered PPG signal as a reference of the heartbeat, and (**e**–**h**) are the singular vectors of the trajectory matrix created from (**c**).

In this section, we present the flow of the SVD+MF method to enhance the weak signal derived from the heartbeat from two baseband signals. Figure 8 shows the signal processing performed to obtain singular vectors from the waveforms obtained in the experiment. Figure 8a,b show the waveforms for 3 s of the baseband signal obtained by the 24 GHz DRS-based measurement system. Figure 8c shows the phase signal of the baseband signal obtained by arctangent demodulation. Figure 8d shows the PPG signal acquired to obtain the reference value of the subject’s heartbeat, and the undesired frequency band was filtered out by the bandpass filter. Finally, Figure 8e,f show the singular vectors of the trajectory matrix formed by the 200-lagged vector in Figure 8c. The time axis in Figure 8d was set to 2 s, which is the length of the singular vector, for a comparison with the singular vector. Here, all y-axes in the figure have been normalized to have mean of 0 and variance of 1.

**Figure 9:**
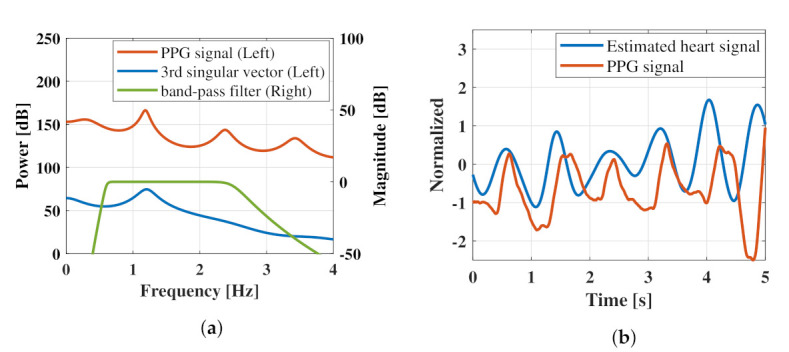
Examples of the heart rate enhancement effect by the proposed technique: (**a**) shows the comparison of the frequency response of the PPG signal, the 3rd singular vector used for heart rate emphasis, and the IIR-type bandpass filter; (**b**) shows the time-domain waveforms of the PPG signal and the heart rate signal estimated by the proposal method.

Although the time-dependent change in the phase signal obtained using Equation (1) is theoretically consistent with the relative vibration of the chest surface and hence may contain weak heartbeat signals, this could not be visually confirmed. As shown by the singular vectors in Figure 8e,f, each vector exhibited a different characteristic shape. Figure 8e shows a trend without a periodic component, whereas Figure 8f,g show signals with a period of approximately 1 s. In the simulation analysis, we concluded that the third singular vector is the most suitable template for extracting the heartbeat. Comparing the third singular vector with the PPG waveform in the figure, the shapes nearly match, although there is a phase shift. This phase shift is thought to be due to the fact that the trajectory matrix where the singular value decomposition is performed is composed of lagged vectors, which are signals with phase shift. To clarify that the calculated third singular vector is effective for heart rate enhancement from the phase signal, an example of the estimation effect of the proposed method on the heart rate signal is presented in Figure 9. Figure 9a shows a comparison of the frequency responses of the third singular vector and PPG signal as a reference for the heartbeat. As the data length of the singular vectors was extremely short (2 s), a multiple signal classification method was used for the analysis of singular vectors and PPG signals. Therefore, its power did not reflect any specific meaning. Figure 9a shows the frequency response of an IIR-type bandpass filter. Figure 9b presents the five-second time-domain waveforms of the PPG signal filtered by the bandpass filter and the phase signal filtered by the third singular vector.

**Figure 10:**
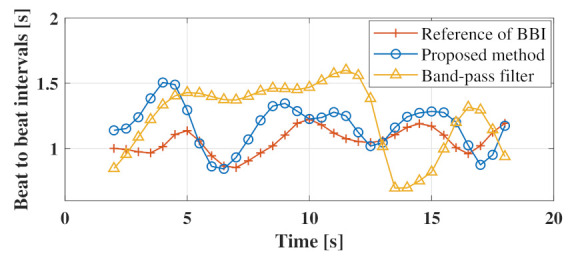
Comparison of heart rate interval plots estimated by the proposed method and bandpass filter.

As shown in Figure 9a, the peak of the spectrum of the third singular vector coincided with one of the peaks of the PPG spectrum at 1.2 Hz. As the matched peaks were caused by the heartbeat, we expected the heartbeat to be emphasized by filtering the phase signal using the third singular vector. Confirming the phase signal with the unnecessary frequency band removed by the third singular vector in Figure 9b, it was confirmed that the peak of the pulse wave obtained via PPG and the peak of the estimated heart rate signal generally coincided well. This fact indicates that the third singular vectors of the trajectory matrix formed from the phase signals can be effective for estimating the heartbeat signal. After the heartbeat waveform in the phase signal was enhanced, a heartbeat-derived peak was detected, and the beat-to-beat intervals were calculated. Figure 10 shows the heartbeat interval plot based on the heart rate signal estimated using the proposed method and bandpass filter. To estimate the heart rate interval, we used the MATLAB function findpeaks to detect the peaks and resampling to convert the sampling frequency to 1 Hz. As shown in Figure 10, the heart rate interval plot estimated using the proposed method was more consistent with the reference than the heart rate interval obtained using the bandpass filter.

### 5.3. Indices for Validating Accuracy of Heart Rate Estimation

To evaluate the accuracy of the heart rate interval estimation, the heart rate information, HR, and SDHI calculated from the heart rate interval were defined as follows:

(14) $$\begin{array}{ccc} HR & = & \frac{1}{N}\sum_{i=1}^{n}\frac{60}{BBI_{i}}\;\left[\mathrm{bpm}\right], \end{array}$$

(15) $$\begin{array}{ccc} SDHI & = & \sqrt{\frac{1}{N}\sum_{i=1}^{n}{\left(BBI_{i}-\mu_{BBI}\right)}^{2}}\;\left[s\right]. \end{array}$$

Here, *N* is the total number of heartbeat intervals in the data interval, $BBI_{i}$ the estimated *i*-th heartbeat interval and $\mu_{BBI}$ the average value of heartbeat interval. The absolute error (AE) was then defined as the accuracy of these indicators and is expressed as follows:

(16) $$\begin{array}{c} AE=|x_{estimated}-x_{reference}|, \end{array}$$

where $x_{estimated}$ is the index estimated by the CW-DRS, and $x_{reference}$ is the index obtained from the reference.

### 5.4. Analysis Results and Discussion of Estimated Cardiac Information

In this section, we calculated the estimation accuracy of the heart rate information using the proposed method for all the data obtained in the experiments. The results from analyzing the data measured in the lying position are shown in Table 3. In this analysis, to confirm the effect of filtering by singular vectors in the proposed method, we compared the results with those obtained using only a bandpass filter. In this analysis, to confirm the effect of filtering by singular vectors in the proposed method, we compared the results with those obtained using only a bandpass filter. The absolute errors of HR, SDHI, and RMSE presented in Table 3 were calculated as follows.

As shown in Table 3, the absolute errors of HR, SDHI, and RMSE were calculated to be 7.16±3.87bpm, 0.14±0.048s, and 0.29±0.056s, respectively, for BPF. In the proposed method, they were reduced to 1.93±1.76bpm, 0.057±0.028s and 0.16±0.047s, respectively. Therefore, the proposed method is considered to be effective for estimating cardiac information for data obtained in the stable supine position, which is assumed to involve only slight body movements.

**Figure 11:**
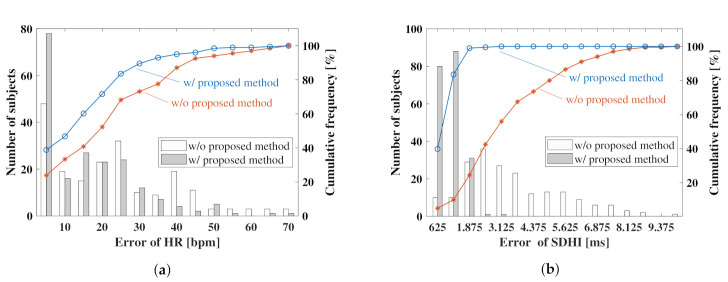
Histograms showing the error of the estimated indices: (**a**) indicates the error distribution of HR, (**b**) represents the error distribution of SDHI.

Next, we analyzed the effectiveness of the proposed method based on the data measured from the subjects in a sitting posture. In this analysis, a histogram was constructed for the absolute error of the calculated cardiac information, as shown in Figure 11, due to the large dataset comprising 212 subjects. Figure 11a shows the error distribution of the HR estimated by the proposed method and BPF. Figure 11b shows the error distribution of the estimated SDHI, where the blue and red lines in each figure represent the cumulative frequency distributions of the data estimated by the proposed method and BPF, respectively.

As shown in Figure 11a, 49 out of 212 subjects (i.e., 23% of the total number of subjects) successfully estimated the HR within 5 bpm by the conventional method, whereas 81 of the 212 subjects (i.e., 37% of the total number of subjects) successfully estimated the HR within 5 bpm using the proposed method. Figure 11b shows that the percentage of subjects who successfully estimated the SDHI within 0.125 s using the proposed method was 83% compared with 10% using the conventional method. The percentage of subjects who successfully estimated each index with high accuracy increased significantly, suggesting that this method is effective for the sitting posture.

Finally, a comparison of the absolute errors of the estimated heart rate information for the two postures is illustrated in Table 4.

Comparing the estimation results of the sitting and supine postures from the table, it was discovered that the error of the sitting posture was larger than that of the lying posture, regardless of the analysis method. This result is speculated to be due to the fact that the subject’s posture was more unstable in the sitting posture than in the lying posture, and that the effect of body motion was superimposed on the baseband signals. Although the estimation accuracy of the proposed method was better than that of the BPF method, it was confirmed that the estimation accuracy of the proposed method was superior to that of the BPF method regardless of the subject’s posture.

## 6. Conclusions

We proposed a novel signal processing algorithm that combines signal decomposition via SVD and signal recovery using an MF to extract weak cardiac signals from the vibration of the chest surface to realize highly accurate heartbeat measurement using CW-DRSs. In this study, we investigated the relationship between the SNR of the demodulated signal and the accuracy of the heartbeat estimated using the proposed method. This was performed by modeling the vibration of the chest surface caused by heartbeat and respiration based on previous studies to determine the optimal parameters for the proposed method. In addition, the accuracy of the proposed method for estimating the heart rate was evaluated by acquiring biological signals in two different postures. Consequently, we observed that the absolute error of the heart rate information estimated using the proposed method decreased significantly compared with that estimated by the BPF in both postures. However, we evaluated data with superimposed body movements that naturally occur in the sitting posture in this study. In addition, regarding the measurement system used in this study, the analog front-end of the sensor back-end was developed in our laboratory. As the amplification of the analog circuit was adjusted in consideration of the measurement environment, a readjustment is necessitated when it is used in another environment.

## Figures and Tables

**Table 1:** Parameters used to generate the respiratory waveform

Parameter	Description	Value
$K_{b}$	Model constants for amplitude adjustment	$10\times 10^{-3}$ (m)
*T*	Period of respiratory waveform	$2.5\times 10^{-3}$ (s)
$T_{i}$	Term of expiration in respiration one wave form	$1.25\times 10^{-3}$ (s)
$T_{e}$	Term of inhalation in respiration one waveform	$1.25\times 10^{-3}$ (s)
τ	Time constant	0.8 (s)

**Table 2:** Parameters used in the simulation of the chest vibration model.

Index	Displacement (mm)	Frequency (bpm)
Heartbeat $m_{heart}\left(t\right)$	0.5	60
Breathing $m_{resp}\left(t\right)$	0.5–20	15
White noise w(t)	0.1	-

**Table 3:** Comparison of heart rate estimation accuracy using bandpass filter on signals obtained from subjects in lying position.

Data	AE of HR (bpm)		AE of SDHI (s)		RMSE (s)	
	BPF	SVD+MF	BPF	SVD+MF	BPF	SVD+MF
1	15.1	5.6	0.138	0.070	0.33	0.19
2	6.6	3.8	0.075	0.067	0.24	0.22
3	7.0	0.80	0.177	0.017	0.24	0.07
4	11.3	3.3	0.224	0.093	0.39	0.16
5	8.0	0.47	0.171	0.046	0.34	0.12
6	1.3	0.60	0.099	0.062	0.20	0.16
7	3.1	0.08	0.077	0.017	0.25	0.11
8	9.5	1.0	0.179	0.030	0.34	0.16
9	5.8	0.67	0.179	0.066	0.30	0.21
10	3.9	2.9	0.112	0.103	0.25	0.21
Total	7.16±3.87	1.93±1.76	0.14±0.048	0.057±0.028	0.29±0.056	0.16±0.047

**Table 4:** Comparison of heart rate estimation accuracy against subject’s posture.

Index	Lying		Sitting	
	BPF	SVD+MF	BPF	SVD+MF
AE of HR (bpm)	7.16±3.87	1.93±1.76	17.1±10.9	9.72±7.86
AE of SDHI (ms)	140±48.1	57.0±28.1	445±222	81.3±24.3
RMSE (ms)	287±56.4	161±47.0	601±548	255±304
